# Developing a core outcome set for the treatment of pregnant women with pregestational diabetes—a study protocol

**DOI:** 10.1186/s13063-020-04910-1

**Published:** 2020-12-11

**Authors:** Oratile Kgosidialwa, Delia Bogdanet, Aoife Egan, Paula M. O’Shea, Linda Biesty, Declan Devane, Fidelma Dunne, On behalf of the INSPIRED group

**Affiliations:** 1 School of Medicine, National University of Ireland Galway, Galway, Ireland; 2 Department of Endocrinology, Mayo Clinic Rochester, Rochester, MN USA; 3 School of Nursing and Midwifery, National University of Ireland Galway, Galway, Ireland; 4 Ireland HRB-Trials Methodology Research Network, National University of Ireland Galway, Galway, Ireland

**Keywords:** Core outcome set, Pregnancy, Pregestational diabetes mellitus, Type 1 diabetes mellitus, Type 2 diabetes mellitus, Intervention, Randomised controlled trials

## Abstract

**Background:**

Pregestational diabetes mellitus (PGDM) is associated with adverse pregnancy outcomes including increased rates of caesarean section birth, macrosomia, congenital malformation, prematurity, admission to the neonatal intensive care unit and stillbirth. As a result, there has been an increase in interventions to improve outcomes in both mother and infant. To date, meaningful comparisons between these studies are limited due to heterogeneity in outcome selection and reporting. The aim of this study is to develop a core outcome set (COS) for randomised controlled trials evaluating the effectiveness of interventions for the treatment of pregnant women with PGDM.

**Methods:**

The study consists of three steps. The first step is a systematic review of the literature to assess outcomes reported in randomised controlled trials assessing the effectiveness of interventions for the treatment of pregnant women with PGDM. The second step is a three round, online Delphi survey to prioritise these outcomes. In this step, stakeholders (including women with PGDM, healthcare workers, researchers and policymakers) will be asked to rank the importance of outcomes for inclusion in the COS using a 9-point Likert type scale. Outcomes that meet the inclusion criteria after completion of the Delphi surveys will be brought to the consensus meeting. The consensus meeting will be the third and final step, where the COS will be finalised. The consensus meeting will include members from each stakeholder group.

**Discussion:**

This paper describes the process used to develop a COS for the reporting of studies evaluating the effectiveness of interventions in pregnant women with PGDM. The COS will enable greater comparison between and information synthesis across RCTs in the treatment of PGDM. In addition, this COS will also help improve trial reporting and minimise research waste by prioritising the collection and reporting of outcomes that matter to all relevant stakeholder groups.

**Trial registration:**

This COS has been registered with the Core Outcome Measures in Effectiveness Trials (COMET) initiative (http://www.comet-initiative.org/studies/details/1425) on the 4th of November 2019. The systematic review component of this study has also been registered with the International Prospective Register of Systematic Reviews (PROSPERO) (https://www.crd.york.ac.uk/prospero/display_record.php?ID=CRD42020173549).

**Supplementary information:**

**Supplementary information** accompanies this paper at 10.1186/s13063-020-04910-1.

## Background

Pregestational diabetes mellitus (PGDM) is defined as diabetes existing prior to pregnancy (including type 1 diabetes mellitus (T1DM) and type 2 diabetes mellitus (T2DM)). The prevalence of PGDM ranges from 1% in a racially diverse community [1] to 4.3% in high-risk populations [2]. The incidence of PGDM continues to increase [3]. There has been a sharp increase in recent years in the incidence of pre-pregnancy T2DM in parallel with the increasing global obesity pandemic especially in newly industrialised and emerging countries [3–6].

PGDM is associated with poor outcomes in women and their infants. These women are more likely to have induction of labour, to have a caesarean birth or have a birth complicated by shoulder dystocia [2, 7, 8]. Additionally, these women are also vulnerable to pregnancy-related comorbid conditions such as pre-eclampsia and pregnancy-induced hypertension [9, 10]. New-borns of women with PGDM are more likely to display macrosomia, be born preterm, to be admitted to the neonatal intensive care unit (NICU), have major congenital malformations or be stillborn [2, 9, 11]. Pre-pregnancy maternal complications such as diabetic retinopathy [12] may also worsen for women with PGDM during pregnancy.

There have been continued efforts in education, technology and pharmacology to improve maternal and infant outcomes in women with PGDM. Technological advances including improved insulin delivery via continuous subcutaneous insulin infusion (CSII) [13], insulin analogues [14] and closed loop systems [15, 16] have and continue to be examined for use by pregnant women with PGDM. Improved glucose testing techniques have also increased in the general diabetes population and some of these techniques have also been examined in pregnant populations, e.g. continuous glucose monitoring (CGM) [17] and flash glucose sensors [18]. Internet and phone applications are emerging tools for self-care for women with PGDM [19, 20].

There is evidence that advancements in technology, education and pharmacology have improved clinical outcomes for women with diabetes in pregnancy [21, 22]. However, the way outcomes are reported often makes it difficult to compare the effects of interventions and robustly synthesise evidence [23]. Thus, we will develop a core outcome set (COS) for randomised controlled trials (RCTs) assessing the effectiveness of interventions for the treatment of pregnant women with PGDM.

A core outcome set is an agreed standardised set of outcomes that should be measured and reported, as a minimum, in all clinical trials in specific areas of health or health care [24]. The aim is to reduce reporting bias and heterogeneity of outcomes in order to support robust evidence synthesis. This process usually involves a wide variety of key stakeholders to ensure that clinically relevant outcomes are identified and reported.

### Scope of the core outcome set

The COS will be applicable to future RCTs evaluating the effectiveness of interventions for the treatment of pregnant women with PGDM. The COS may also be useful for studies beyond trials and for routine clinical practice.

It is known that pre pregnancy care (PPC) as an intervention in women with PGDM improves both maternal and neonatal outcomes [25]. PPC is outside the scope of this work and will not be addressed in this project. A COS for PPC as an intervention has already been developed [26].

### Study work packages

**Fig. 1:**
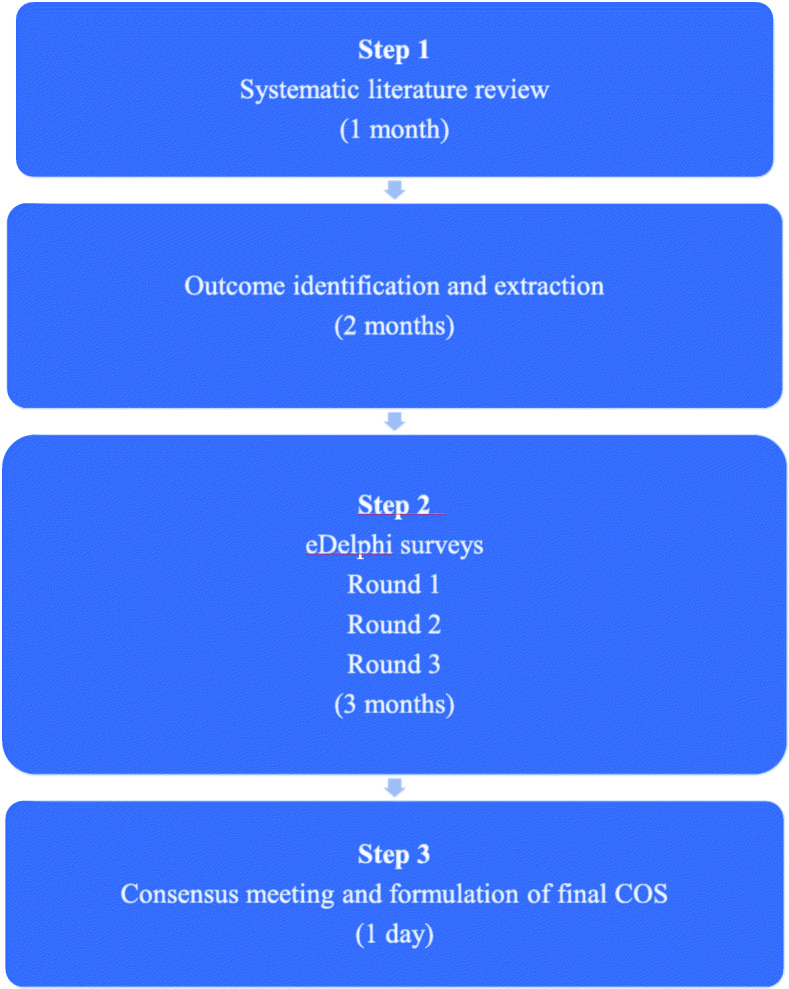
Flowchart of work schedule. COS, core outcome set

This study has 3 components (Fig. 1):

1. A systematic literature review to identify a list of all outcomes reported in prior or ongoing RCTs of interventions for the treatment of pregnant women with PGDM.
2. A three-round e-Delphi survey where key stakeholders will prioritise these outcomes.
3. A consensus meeting where a list of core outcomes will be finalised and form the COS. The final core outcome set will be published in a scientific journal

## Methods

Preparation of this protocol is in line with the COS-STAP Statement recommendations which gives guidance on items considered essential in a COS protocol [27]. Our methodology is similar to previous work our group has carried out in development of COS in other areas of maternal diabetes [28–30].

To date, we are not aware of any published COS of RCTs evaluating the effectiveness of interventions in pregnant women with PGDM. This COS has been registered with the Core Outcome Measures in Effectiveness Trials (COMET) initiative. (http://www.comet-initiative.org/studies/details/1425). The systematic review component of this study has also been registered with the International Prospective Register of Systematic Reviews (PROSPERO) (https://www.crd.york.ac.uk/prospero/display_record.php?ID=CRD42020173549).

### Ethics

Ethical approval for this study was granted by the Clinical Research Ethics Committee, Galway University Hospitals, Galway, Ireland (Ref: C. A 2293).

### Step 1: Systematic review and identification of previously reported outcomes

#### Systematic review question

What are the outcomes reported in RCTs evaluating the effectiveness of interventions in pregnant women with PGDM?

#### Population

Pregnant women with PGDM.

#### Interventions

Any intervention offered to the pregnant woman with PGDM will be included. Interventions can be broadly categorised into technological, pharmacological, lifestyle and educational.

#### Types of studies

##### Randomised trials

Trials assessing outcomes in both PGDM and GDM in the same study will also be included. Longitudinal follow-up studies and secondary analysis of clinical trials will be excluded. Reviews, reports of conference proceedings or abstracts where there is no complete description of the trial methodology will also be excluded.

#### Search strategy and information sources

A search strategy will be formulated with the assistance of a librarian at the National University of Ireland, Galway (NUI Galway). A PICO format will be used to guide the search strategy (Table 1). A combination of keywords and Medical Subject Headings (MeSh) terms will be used to search for specific concepts, which will then be combined using Boolean operators to formulate the final search strategy.

**Table 1:** Search strategy selection criteria of RCTs assessing outcomes of treatment interventions in pregnant women with PGDM

Population	Pregnant women with pregestational diabetes
Intervention	Any intervention including education, lifestyle, pharmacology and technology
Comparator	Any comparator or control
Outcome	Any measured or reported outcome

*RCTs* randomised controlled trial, *PGDM* pregestational diabetes mellitus

The following databases will be searched: CENTRAL (via the Cochrane Library), Web of Science (WOS), MEDLINE (via OVID platform), Cumulative Index of Nursing and Allied Health Literature (CINAHL) (via EBSCO host platform) and Embase. In addition, ClinicalTrials.gov will be searched for ongoing trials. The references of clinical trials evaluating interventions in the treatment of pregnant women with PGDM will be checked for studies not captured in the search. Publications are restricted to those in English. There will be no time restrictions applied to the search strategy; however, the final search will be completed by the end of January 2020. A sample search strategy is shown in the supplementary material (see supplementary file 1).

#### Study assessment

Studies deemed suitable for inclusion will be identified from the search using the predetermined inclusion criteria. Two independent reviewers (OK and DB) will screen titles and abstracts of the selected studies to ensure eligibility. Full-text papers of selected studies will be reviewed by both reviewers prior to final decision regarding inclusion. Disagreements will be resolved through discussion and recourse to a third author (FD) if necessary.

#### Data extraction

Outcomes for potential inclusion into the COS will be extracted from the ‘methods’ and ‘results’ section of each paper. We will then assess how each outcome was defined, the instruments or indicators used to measure the outcome and the time points or periods of outcome measurement. In addition, a data extraction template consisting of the following parameters will be formulated; authors, the study design, the condition of interest (T1DM, T2DM or both), journal and year of publication.

Two independent reviewers (OK and DB) will assess the articles independently, review outcomes together and ensure that all outcomes have been identified and included.

#### Data analysis

All eligible studies will be tabulated. Outcomes identified for inclusion will be listed and defined. Any differences in definitions of outcomes will be noted. All outcomes identified will be categorised into appropriate domains by the authors. The Study Advisory Group (SAG), involving healthcare workers, researchers and women with a history of PGDM will then review the outcomes and outcome domains and assess suitability of grouping of outcomes and titles of the domains.

### Step 2: eDelphi survey involving key stakeholders

#### Stakeholders

Stakeholders in this COS will include women with a history of PGDM, clinicians, researchers and policy makers. The research team involved in this COS have diverse experience in both clinical maternal diabetes and COS research. There are 3 main broad stakeholder groups.

##### Group 1: Service users

Women with PGDM who have been pregnant previously or are currently pregnant will be invited to participate. These women will be identified from a variety of sources. Women currently attending our antenatal or general diabetes clinics will be invited to participate face to face, via post or email. Clinical leads in other hospitals will also be contacted via email to invite women attending their service to participate. In addition, known diabetes service user group managers will be emailed to invite women to participate.

##### Group 2: Clinicians and researchers

An international cohort of clinicians and researchers will also be invited to participate in the COS. Clinicians will be invited to participate from all areas of care associated with pregnant women. This will include diabetologists, diabetes nurse specialists, midwives, dieticians, obstetricians, neonatologists, paediatricians, clinical biochemists, general practitioners, practice nurses, occupational therapists and physiotherapists. Clinical leads of both national and international organisations involved in the treatment and/or research of women with PGDM will be identified and recruited. International organisations of specific interest include the Diabetic Pregnancy Study Group (DPSG), a study group of the European Association for the Study of Diabetes (EASD), the International Association of Diabetes and Pregnancy Study Groups (IADPSG), the International Federation of Gynaecology and Obstetrics (FIGO), the European Board and College of Obstetrics and Gynaecology (EBCOG), the American Diabetes Association (ADA) the Canadian Diabetes Association (CDA) and the Australian Diabetes in Pregnancy Society (ADIPS). Clinical leads will be recruited via email and encouraged to invite participants from their pregnancy care teams.

##### Group 3: Policy makers

National and international policy makers from the Health Service Executive (HSE), International Diabetes Federation (IDF) and World Health Organization (WHO) will be invited.

All potential participants will be sent an invitation to participate in the study via email. The invitation will be in plain English where possible. Where medical terms are unavoidable, a lay description of the word or phrase will be given in parenthesis. This invitation will include the synopsis and aim of the study and a link to the online survey. Prior to commencing the survey at registration, participants will be explicitly required to provide informed consent. All those receiving the online invitation will be encouraged to also forward it to anyone they deem to have expertise in any field of maternal diabetes.

#### Online international eDelphi surveys

A Delphi survey will be used to develop a consensus of the most important outcomes of interventions in women with PGDM identified from the systematic review. The Delphi is a group facilitation technique that seeks to obtain consensus on the opinions of ‘experts’ through a series of structured questionnaires (commonly referred to as rounds) [31]. The rounds are a repetitive multistage process designed to combine opinions into group consensus [31].

After completion of the systematic review described in step 1, a long list of potential outcomes will be available. Clinical terms will be explained using plain English to help service users better understand outcomes. Outcomes will be grouped into domains. To avoid potentially weighting outcomes in the order with which they are displayed and introducing bias, outcomes will be randomly listed within the specific domains.

Participants will rank importance of potential outcomes for inclusion in the COS using a 9-point Likert type scale with score l representing an outcome of least importance and 9 representing outcome of critical importance. This scale was formulated by the Grading of Recommendations Assessment, Development and Evaluation (GRADE) Working Group in order to help facilitate the ranking of outcomes according to their importance and has been used widely in the process of COS development [32].

A prespecified consensus criteria which was previously used by our group, will be used to either include or exclude outcomes at the end of each round [33, 34] (Table 2). In brief, ‘Consensus in’ for any outcome will be defined as ≥ 70% participants scoring 7 to 9 and < 15% scoring 1 to 3. ‘Consensus out’ will be defined as ≤ 50% participants scoring 7–9 in each stakeholder group and will be excluded. Outcomes that do not meet any of these criteria were labelled as ‘no consensus’.

**Table 2:** Delphi consensus definition

Consensus decision	Definition	Scoring
Consensus in	Consensus that outcome should be included in the COS	≥ 70% participants scoring 7 to 9 AND < 15% scoring 1 to 3.
Consensus out	Consensus that outcome should be excluded in the COS	≤ 50% participants scoring 7–9 in each stakeholder group
No consensus	Uncertainty about importance of the outcome	Anything else not fitting criteria above for ‘consensus in’ or ‘consensus out’.

*COS* core outcome set [34]

After each round of questions, an anonymous summary of the responses and participants’ own individual response will be reported back to the group. At this stage, participants may choose to keep their original responses or change their stance in the following round.

##### Round 1

Upon consenting to participate in the online questionnaire as described above, participants will be requested to record their name, gender, country of residence, email address and centre they are affiliated with. In addition, they will be asked to identify the stakeholder group and subgroup to which they belong. Participants will be required to complete the survey within 4 weeks with up to two reminder emails to complete the questionnaire sent at least a week prior to closing the survey to reduce attrition rates.

Participants will be invited to include any other outcomes that they think may have been missed which will be included in subsequent eDelphi survey sent to participants. Participants will be limited to adding two potential outcomes only. Outcomes will be included in the subsequent eDelphi round if at least two participants have listed it as a possible outcome.

##### Round 2

The round 2 instrument will consist of all outcomes brought forward from round 1. Additional unique outcomes suggested by at least two participants in round 1 will be included in the round 2 survey with the outcomes progressing from round 1. All participants who completed round 1 will be invited to take part in round 2 of the eDelphi. All outcomes from round 1 including any new additional outcomes meeting the inclusion criteria will be included in round 2. As in round 1, outcomes will be presented with a 9-point Likert type scoring system. Participants will be presented with findings from the round 1 survey, i.e. the number of participants taking part, the proportion of people scoring each rating point on the Likert scale for each stakeholder group and the group overall and how their own scores compare to the rest of the group.

Based on the feedback provided to participants after round 1, they will be asked to rate the outcomes again using the same 9-point scale as used in round 1. This will give participants the opportunity to change their scoring on outcomes based on the knowledge of what they and their stakeholder group scored in round 1.

##### Round 3

The round 3 instrument will consist of all outcomes classified as ‘consensus in’ brought forward from round 2. Only participants who completed round 1 and 2 will be invited to participate in round 3. Participants will be provided with feedback as in round 2 and invited to rate each outcome. All outcomes classified as ‘consensus in’ (Table 2) will be brought forward to the consensus meeting.

### Step 3: Consensus meeting

The final stage of this work will be an international consensus meeting with members of the relevant stakeholder groups to discuss, review and vote on a final COS. We plan to have at least 20 participants, with members from each stakeholder group participating to ensure maximum diversity of the demographic representation. Participants will be sent the results of round 3 of the Delphi at least 2 weeks prior to the meeting to allow time for reflection of their answers and those of others.

This meeting will be chaired by an experienced, non-voting facilitator. After a prior discussion on each outcome, anonymous electronic voting will take place whereupon consensus will be defined as per previously stated in the eDelphi survey. Prior to the conclusion of the consensus, the recommended list of outcomes will be reviewed and finalised.

#### Dissemination and implementation

Upon completion of the consensus meeting and agreement on the final COS, a manuscript will be prepared in compliance with the CO-STAP recommendations [27], published and disseminated. To encourage researchers and clinicians to use the COS, we aim to present this work at national and international conferences.

## Conclusion

Currently, there is no published COS for RCTs evaluating the effectiveness of interventions in pregnant women with PGDM. The COS will enable greater comparison between and information synthesis across RCTs in the treatment of PGDM. In addition, this COS will help improve trial reporting and minimise research waste by prioritising the collection and reporting of outcomes that matter to all relevant stakeholder group.

### Study status

The study is currently at the systematic literature review stage. Current protocol; version 1 dated 27 April 2020.

## Supplementary information

**Additional file 1.** Sample search strategy.

## Data Availability

All authors will have access to data.

## Abbreviations

PGDM: Pregestational diabetes
COS: Core outcome set
RCT: Randomised controlled trial
COMET: Core Outcome Measures in Effectiveness Trials
PROSPERO: International Prospective Register of Systematic Reviews
T1DM: Type 1 diabetes mellitus
T2DM: Type 2 diabetes mellitus
NICU: Neonatal intensive care unit
CSII: Continuous subcutaneous insulin infusion
CGM: Continuous glucose monitor
PPC: Pre-pregnancy care
COS-STAP: Core Outcome Set-STAndardised Protocol Items
WOS: World of science
CINAHL: Cumulative Index of Nursing and Allied Health Literature
SAG: Study Advisory Group
DPSG: Diabetic Pregnancy Study Group
EASD: European Association for the Study of Diabetes
IADPSG: International Association of Diabetes and Pregnancy Study Groups
FIGO: International Federation of Gynaecology and Obstetrics
EBCOG: European Board and College of Obstetrics and Gynaecology
ADA: American Diabetes Association
CDA: Canadian Diabetes Association
ADIPS: Australian Diabetes in Pregnancy Society
HSE: Health Service Executive
IDF: International Diabetes Federation
WHO: World Health Organization
GRADE: Grading of Recommendations Assessment, Development and Evaluation
