# Supplementary material for: Developing a core outcome set for the treatment of pregnant women with pregestational diabetes—a study protocol
Source: Trials. 2020 Dec 11;21:1017. doi: 10.1186/s13063-020-04910-1 (PMC7730783; doi:10.1186/s13063-020-04910-1)
Supplement: Supplementary file 1 — Additional file 1. Sample search strategy. [file 13063_2020_4910_MOESM1_ESM.docx]

**Sample search strategy**

EMBASE (via EBSCOHOST platform) (Up to 16/1/20)

| No. | Query | Results | Date |
| --- | --- | --- | --- |
| #11 | ((('pre-gestational diabetes':ti,ab,kw OR 'pregestational diabetes':ti,ab,kw) OR (('preexisting diabetes':ti,ab,kw OR 'pre-existing diabetes':ti,ab,kw) AND pregnan*:ti,ab,kw) OR (('type 1 diabetes':ti,ab,kw OR t1dm:ti,ab,kw OR 'insulin dependent diabetes':ti,ab,kw OR 'type 2 diabetes':ti,ab,kw OR t2dm:ti,ab,kw OR 'insulin independent diabetes':ti,ab,kw) AND pregnan*:ti,ab,kw)) AND (intervention:ti,ab,kw OR therapy:ti,ab,kw OR treatment:ti,ab,kw OR education:ti,ab,kw)) AND 'human'/de AND ('randomized controlled trial'/de OR 'randomized controlled trial (topic)'/de) | 283 | 16 Jan 2020 |
| #10 | ((('pre-gestational diabetes':ti,ab,kw OR 'pregestational diabetes':ti,ab,kw) OR (('preexisting diabetes':ti,ab,kw OR 'pre-existing diabetes':ti,ab,kw) AND pregnan*:ti,ab,kw) OR (('type 1 diabetes':ti,ab,kw OR t1dm:ti,ab,kw OR 'insulin dependent diabetes':ti,ab,kw OR 'type 2 diabetes':ti,ab,kw OR t2dm:ti,ab,kw OR 'insulin independent diabetes':ti,ab,kw) AND pregnan*:ti,ab,kw)) AND (intervention:ti,ab,kw OR therapy:ti,ab,kw OR treatment:ti,ab,kw OR education:ti,ab,kw)) AND 'human'/de | 2645 | 16 Jan 2020 |
| #9 | (('pre-gestational diabetes':ti,ab,kw OR 'pregestational diabetes':ti,ab,kw) OR (('preexisting diabetes':ti,ab,kw OR 'pre-existing diabetes':ti,ab,kw) AND pregnan*:ti,ab,kw) OR (('type 1 diabetes':ti,ab,kw OR t1dm:ti,ab,kw OR 'insulin dependent diabetes':ti,ab,kw OR 'type 2 diabetes':ti,ab,kw OR t2dm:ti,ab,kw OR 'insulin independent diabetes':ti,ab,kw) AND pregnan*:ti,ab,kw)) AND (intervention:ti,ab,kw OR therapy:ti,ab,kw OR treatment:ti,ab,kw OR education:ti,ab,kw) | 2879 | 16 Jan 2020 |
| #8 | intervention:ti,ab,kw OR therapy:ti,ab,kw OR treatment:ti,ab,kw OR education:ti,ab,kw | 8135469 | 16 Jan 2020 |
| #7 | ('pre-gestational diabetes':ti,ab,kw OR 'pregestational diabetes':ti,ab,kw) OR (('preexisting diabetes':ti,ab,kw OR 'pre-existing diabetes':ti,ab,kw) AND pregnan*:ti,ab,kw) OR (('type 1 diabetes':ti,ab,kw OR t1dm:ti,ab,kw OR 'insulin dependent diabetes':ti,ab,kw OR 'type 2 diabetes':ti,ab,kw OR t2dm:ti,ab,kw OR 'insulin independent diabetes':ti,ab,kw) AND pregnan*:ti,ab,kw) | 8936 | 16 Jan 2020 |
| #6 | ('type 1 diabetes':ti,ab,kw OR t1dm:ti,ab,kw OR 'insulin dependent diabetes':ti,ab,kw OR 'type 2 diabetes':ti,ab,kw OR t2dm:ti,ab,kw OR 'insulin independent diabetes':ti,ab,kw) AND pregnan*:ti,ab,kw | 7276 | 16 Jan 2020 |
| #5 | ('preexisting diabetes':ti,ab,kw OR 'pre-existing diabetes':ti,ab,kw) AND pregnan*:ti,ab,kw | 680 | 16 Jan 2020 |
| #4 | pregnan*:ti,ab,kw | 660805 | 16 Jan 2020 |
| #3 | 'type 1 diabetes':ti,ab,kw OR t1dm:ti,ab,kw OR 'insulin dependent diabetes':ti,ab,kw OR 'type 2 diabetes':ti,ab,kw OR t2dm:ti,ab,kw OR 'insulin independent diabetes':ti,ab,kw | 268667 | 16 Jan 2020 |
| #2 | 'preexisting diabetes':ti,ab,kw OR 'pre-existing diabetes':ti,ab,kw | 1670 | 16 Jan 2020 |
| #1 | 'pre-gestational diabetes':ti,ab,kw OR 'pregestational diabetes':ti,ab,kw | 1452 | 16 Jan 2020 |

COCHRANE Library (Up to 19/1/20)

| ID | Search | Hits |
| --- | --- | --- |
| #1 | MeSH descriptor: [Pregnancy in Diabetics] explode all trees | 303 |
| #2 | ("type 1 diabetes" OR T1DM OR "insulin dependent diabetes" OR "type 2 diabetes" OR T2DM OR "insulin independent diabetes" OR "pregestational diabetes" OR "pre-gestational diabetes" OR "preexisting diabetes" OR "pre-existing diabetes"):ti,ab,kw | 42407 |
| #3 | #1 AND #2 | 98 |
| #4 | (pregnan*):ti,ab,kw | 60455 |
| #5 | ("type 1 diabetes" OR T1DM OR "insulin dependent diabetes" OR "type 2 diabetes" OR T2DM OR "insulin independent diabetes" OR "preexisting diabetes" OR "pre-existing diabetes"):ti,ab,kw | 42346 |
| #6 | #4 AND #5 | 1300 |
| #7 | (Intervention OR treatment OR therapy OR management OR education):ti,ab,kw | 1117350 |
| #8 | #6 AND #7 | 1158 |
| #9 | #3 AND #7 | 85 |
| #10 | #8 OR #9 | 1164 |
| #11 | ("randomized controlled trial"):ti,ab,kw | 474488 |
| #12 | #10 AND #11 | 372  Trials (333)  Reviews (39) |
